# The importance of pre-epiglottis space invasion in the treatment planning of larynx and hypopharynx cancer

**DOI:** 10.1016/S1808-8694(15)30754-0

**Published:** 2015-10-19

**Authors:** Abrão Rapoport, Renato Assayag Botelho, Ricardo Pires de Souza, Saulo Montenegro Cavalcanti, Sérgio Furlam, Olger de Souza Tornin, Tânia Regina Bastos Souza

**Affiliations:** 1 Livre docente habilitation, USP Medical School, Technical Director of the Health Department – Heliopolis Hospital; 2 Master's degree, Health Sciences graduate course, Heliopolis Hospital, HOSPHEL, Sao Paulo. Radiologist in the Image Diagnosis Department, Heliopolis Hospital, HOSPHEL, Sao Paulo; 3 Doctor, Radiology Department, Sao Paulo Federal University, Radiologist in the Image Diagnosis Department, Heliopolis Hospital, HOSPHEL, Sao Paulo; 4 Resident, Image Diagnosis Department, Heliopolis Hospital, HOSPHEL, Sao Paulo; 5 Resident, Image Diagnosis Department, Heliopolis Hospital, HOSPHEL, Sao Paulo; 6 Master's degree, Health Sciences graduate course, Heliopolis Hospital, HOSPHEL, Sao Paulo, Radiologist in the Image Diagnosis Department, Heliopolis Hospital, HOSPHEL, Sao Paulo; 7 Master's degree, Head & Neck Surgery Department, Santa Casa de Misericordia, Sao Paulo. Surgeon, Otorhinolaryngology and Head & Neck Surerm Department, Heliopolis Hospital, HOSPHEL, Sao Paulo

**Keywords:** cancer, pre-epiglottisc space, pharynx, larynx

## Abstract

The involvement of pre-epiglottis space can change the indication for partial laryngeal resection.

**Aim:**

The aim of this study was to evaluate inter-observer and intra-observer agreement by means of computed tomography analysis regarding the involvement of the pre-epiglottis space (PES) from carcinoma of the upper aerodigestive tract and its relation with therapeutic planning.

**Materials and Methods:**

Retrospective study of ninety-five computed tomography exams of patients with squamous cell carcinoma, from 1990 to 2004, were selected and evaluated; 87 were males and eight females, with ages ranging from 32 to 73 years. Imaging results were analyzed twice by three radiologists, individually, without any previous knowledge of the clinical stage. No patient had received any previous treatment up to the moment of imaging examination, such as surgery, chemotherapy or radiotherapy. All the cases were confirmed by biopsy. Information was obtained from the medical charts.

**Results:**

Kappa Index was calculated by assessing agreement between the three observers. We obtained substantial to almost perfect levels of agreement.

**Conclusions:**

After a general Kappa Index of 0.72, the results suggest a substantial agreement in the involvement of the PES by means of computed tomography analysis.

## INTRODUCTION

The preepiglottic space, also called Boyer's space,^1^ is an areolar triangular structure containing mostly adipose tissue, but also some elastic fibers, collagen fibers and lymphatic ducts. It is an anterior space located between the ventral surface of the epiglottis (posterior margin) and the anterior laryngeal area, extending into the lower portion of the hyoid bone until the midportion of the thyroid cartilage. The preepiglottic space is defined superiorly by the hyoepiglottic ligament, anteriorly by the thyrohyoid membrane and the thyroid cartilage, and posteriorly by the epiglottal cartilage and the thyroepiglottic ligament, which defines the posterior and inferior interface between the preepiglottic space and the paraglottic space.^2^, ^3^, ^4^, ^5^, ^6^ The postero-superior limit between these spaces is evident in most studies. The preepiglottic space, however, is separated from the paraglottic space by collagen division membranes and elastic fibers extending from the tip of the epiglottis to the tip of the laryngeal prominence.^7^

Tumor staging and treatment may be altered after establishing neoplastic cell preepiglottic space invasion. Inadequate blood supply to the preepiglottic space may lead to necrosis of the central area of the tumor, which may explain its resistance to radiotherapy and the superior cure rates obtained with surgery.^5^

Computed tomography (TC) is indicated, among various diagnostic methods, as the main approach to assess tumor invasion of the preepiglottic space; it is more accessible and at least as sensitive as magnetic resonance imaging (MRI).^8^ Most of the studies on this topic, however, were done on small samples in which there was poor correlation with histopathology.^9^

New studies with larger series are needed, given the uncertainties around the true efficacy of CT in evaluating the preepiglottic space. It is essential to verify the tomographic signs that suggest invasion of the preepiglottic space to define the validity of these studies for the daily medical and radiological routine. This evaluation was done in the current study by analyzing statistically the results of intra- and interobservations between three radiologists, where reliability criteria for assessing invasion of the preepiglottic space and their repercussion on therapy for oropharyngeal lesions (tonsillar and base of the tongue) were established.

## METHOD

**Figure 1:**
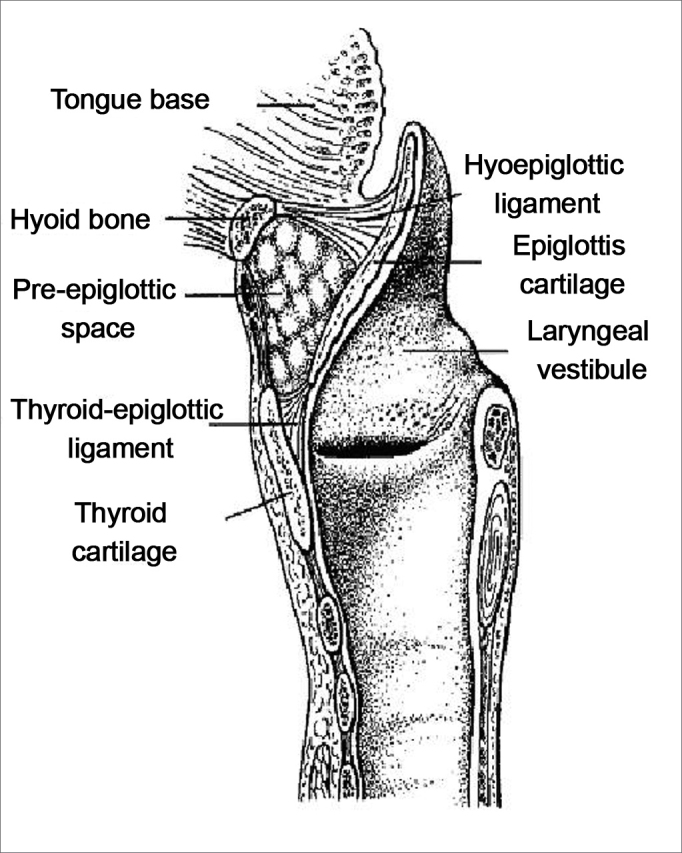
Schematic representation of the sagittal anatomy of the larynx, demonstrating the preepiglottic space and its limits (modified from Dursun).

A retrospective non-randomized study (approved by the Research Ethics Committee, number 401) was undertaken, in which computed tomographic exams of the cervical region (done between 1990 and 2004) of 95 patients diagnosed with epidermoid carcinoma of the upper aerodigestive tract were evaluated. The primary tumor sites were the larynx, the tonsillar area, the hypopharynx, and the base of the tongue (Frame 1). Radiological signs of invasion of the preepiglottic space are shown in Figure 1. There were eight (8.4%) female patients and 87 male patients (91.6%) aged from 32 to 73 years, mean – 55.6 years, median – 57 years. Smoking was reported in 71 patients (74.7%) and drinking alcohol was reported in 64 patients (67.6%); all of those that consumed alcohol were also smokers.

**Frame 1:** Assessment of interobserver agreement according to the calculated Kappa index.

KAPPA INDEX	AGREEMENT
< 0,20	NEGLIGIBLE
0,21 – 0,40	MINIMAL
0,41 – 0,60	MODERATE
0,61 – 0,80	GOOD
0,81 – 1,0	EXCELLENT

Inclusion criteria were: the presence of primary laryngeal tumors, and tumors of the base of the tongue, the tonsillar area and the hypopharynx; CT films for interpretation and anatomical and pathological interpretation; and a pathological and anatomical diagnosis of epidermoid carcinoma. Exams done after radiotherapy (18 cases) were excluded from this study. There were, finally, 95 patients, of which 41 (43.2%) had laryngeal tumors, 24 (25.2%) had tonsillar tumors, 17 (17.9%) had hypopharyngeal tumors, and 13 (13.7%) had tumors on the base of the tongue.

### Computed tomography technique

Three CT devices were used: a Somation Emotion (Siemens), a TCT – 500S (Toshiba Medical Incorporation) and a CT – MAX (General Electric Medical Systems).

Patients were placed in dorsal decubitus; image acquisition was in the axial plane from the suprasellar area to the superior portion of the sternoclavicular joint; the gantry was angled perpendicular to the aerodigestive column; section thickness and increment were 05 mm.

Endovenous iodine contrast (1.0 to 2.0 mL/Kg, 60% and 76% concentration) was injected in all of the patients.

The observers selected for this study had about four years experience in head and neck radiology. Their master's degree dissertations were related to the topic interobserver agreement in head and neck radiology; observer number two was a graduate student. Exams were evaluated twice at an at least 30-day interval between each assessment. No physical examination, laryngoscopic or histopathological data was provided to the observers. Signs of preepiglottic space involvement were fat stranding, local mass effect, and direct involvement of the lesion characterized by the presence of tumor with a similar density to that of soft tissues in the area under study.

Following CT exam interpretation, the Kappa (k) index was used to assess interobserver agreement based on the agreement criteria shown on Frame 1; the significance level was p = 0.05 at a 95% confidence interval.

The EPIDAT software – Epidemiological Analysis of Tabulated Data, version 1.0, 1994, Junta of Galicia, Panamerican Health Organization – was used for the statistical analysis.

## RESULTS

The three observers were analyzed jointly, testing the null hypothesis (no agreement between observers – Kappa = 0) against the alternative hypothesis (agreement -Kappa > 0) for computer tomographic signs suggesting neoplastic invasion of the preepiglottic space.

Estimated Kappa coefficient results, the 95% confidence interval, the p value associated with the test and the classification of agreement, are presented in Table 1, Tabela 2, Table 3, Table 4, Table 5.

**Table 1:** Kappa coefficients for the first interobserver evaluation.

Observers	Kappa	Confidence Interval	p	Agreement
1 and 2	0,631	0,441–0,82	<0,001	Boa
1 and 3	0,627	0,432–0,822	<0,001	Boa
2 and 3	0,739	0,539–0,94	<0,001	Boa

Kappa mean: 0.665

Agreement: Good

**Tabela 2:** Coeficientes Kappa para a segunda avaliação interobservadores.

Observers	Kappa	Confidence Interval	p	Agreement
1 and 2	0,724	0,53–0,917	<0,001	Good
1 and 3	0,664	0,464–0,863	<0,001	Good
2 and 3	0,716	0,519–0,913	<0,001	Good

Kappa mean: 0.701

Agreement: Good

**Table 3:** Kappa coefficients for the second interobserver evaluation.

Observers	Kappa	Confidence Interval	p	Agreement
1	0,853	0,654–1,0	<0,001	Excellent
2	0,91	0,709–1,0	<0,001	Excellent
3	0,85	0,649–1,0	<0,001	Excellent

Kappa mean: 0.871

Agreement: Excellent

**Table 4:** Distribution of patients according to preepiglottic space involvement relative to all of the readings of cases clinically staged as T1 and T2.

			Observations			
	1st reading obs 1	1st reading obs 2	1st reading obs 3	2nd reading obs 1	2nd reading obs 2	2nd reading obs 3
+	9	11	16	17	12	12
–	20	18	13	12	17	17
Total	29	29	29	29	29	29

Kappa = 0.641 (Confidence Interval: 0.547–0.735);

obs – observer

Agreement: Good

**Table 5:** Distribution of patients according to preepiglottic space involvement relative to all of the readings of cases clinically staged as T3 and T4.

			Observations			
	1st reading obs 1	1st reading obs 2	1st reading obs 3	2nd reading obs 1	2nd reading obs 2	2nd reading obs 3
+	33	38	42	45	42	41
–	33	28	24	21	24	25
Total	66	66	66	66	66	66

Kappa = 0.748 (Confidence Interval: 0.685–0.81);

obs – observer

Agreement: Good

Value of p<0.0001 represent rejection of the null hypothesis at a 5% significance level.

## DISCUSSION

Oroscopy and laryngoscopy assess the mucosal surface of the base of the tongue, making it possible to evaluate the extension of a neoplasm on that site. Sub-mucosal and deep tumor extension, however, cannot be assessed in this way. Invasion of these anatomical sites needs to be correctly evaluated so that treatment of malignant oropharyngeal, hypopharyngeal and laryngeal neoplasms may be adapted accordingly. When the preepiglottic space is invaded, the supraglottic portion of the larynx is resected if the tumor is located in the base of the tongue, while a total laryngeal resection is indicated (rather than partial resection) if the tumor is located in the hypopharynx or larynx.

Imaging tests have been added to other methods used in staging these tumors, given the difficulty or impossibility of obtaining information about deep spaces and structures in the neck by oroscopy and laryngoscopy, and the need to precisely identify tumor extension. In the past, plain and contrasted radiography had a role in tumor staging. Following the development of third generation CT equipment, those exams became obsolete; currently, only MRI compares with CT in these cases. MRI, however, is more expensive and less available than CT, and has not been shown in the literature to be superior in this context.^8^^,^^11^^,^^12^

Published papers disagree on the efficacy of CT in characterizing preepiglottic space invasion. Comparisons between CT and postoperative results in assessing laryngeal carcinoma extension to the preepiglottic space^6^^,^^13^ have shown a sensitivity of 86.6% and 84%, and a specificity of 73.3% and 75%. One of the studies included 40 cases, aiming to correlate CT and MRI assessments of the preepiglottic space for identifying tumor invasion with anatomic and pathological findings; it concluded that CT had excellent sensitivity and specificity rates, comparable to MRI.^8^ Other studies,9 however, found that most of the reviewed papers had insufficient samples and poor correlation with anatomy and pathology, concluding that further trials were needed to assess the true capability of CT in detecting tumor invasion of the preepiglottic space.

To undertake studies that correlate CT results with anatomic and pathological data on tumor invasion of the preepiglottic space, it is first necessary to have studies that assess the reproducibility of those CT results. If reproducibility is inadequate, there is no practical sense in carrying out studies that include correlation of anatomical and pathological data, as there will be no consensus between professionals on radiological criteria that would suggest preepiglottic invasion.^14^ If consensus is lacking, other methods, such as fine needle aspiration, which has been shown to be effective in such cases,^2^^,^^15^ would be preferable to evaluate tumor invasion of the preepiglottic space.

In the present study, three radiologists evaluated 95 computed tomographs of the neck in two separate occasions; they had no access to information from the patient's charts or from readings done previously. Analysis of the resulting data is shown on Table 1, Tabela 2, Table 3, Table 4, Table 5. Kappa index calculations were made to verify:

• observer agreement in the first reading
• observer agreement in the second reading
• observer agreement in both readings
• observer agreement in both readings for tumors clinically classified as T1 and T2
• observer agreement in both readings for tumors clinically classified as T3 and T4

The choice in favor of this calculation method aimed at simulating various situations in which Kappa indices below 0.6 could be found. According to cited agreement criteria,10 Kappa value below 0.6 indicate moderate, minimal or negligible interobserver agreement, depending on the specific Kappa value. The sample was divided into two groups, according to the clinical staging, to bring up any sample selection bias. The rationale was that invasion by the larger T3 and T4 classified tumors would be more easily interpreted, and that most of the divergences in the literature concern clinically staged T1 and T2 cases. Without this division, there might have been higher general agreement due to a larger number of T3 and T4 cases, which would not be reproduced in radiological practice. According to the literature, a correct assessment of tumor invasion of the preepiglottic space is needed most in clinically classified T1 and T2 tumors; identifying tumor invasion of the preepiglottic space in advanced cases does not significantly impact the medical strategy.^9^ Radiotherapy might be preferred to surgery in initial cases (T1 and T2) to preserve the larynx.

Agreement rates were found to be good or excellent in the statistical analysis, with Kappa values higher than 0.6 in all of the calculation conditions mentioned above (general Kappa = 0.721). These results show that there is good reproducibility in the interpretation of radiological data suggesting preepiglottic space invasion. There was similar agreement in another published paper,16 in which the interobserver analysis of 39 cases resulted in a general Kappa index = 0.74 (varying from 0.61 to 0.80).

An assessment of 14 base of the tongue carcinoma cases resulted in a general Kappa index = 0.5 (varying from 0.5 to 0.6), meaning moderate agreement.^17^ Analysis by tomography of 25 tonsillar epidermoid carcinoma patients resulted in a general Kappa index between 0.21 and 0.40 (minimal agreement).^18^ The authors stated that such low Kappa values could be a consequence of falsely interpreting the hyoepiglottic ligament as tumor invasion of the preepiglottic space17 or the absence of sagittal tomographic sections.^18^ A further possibility not presented by those authors, and that might have influenced the results, could have been the limited sample size (number of exams); in such cases, interobserver disagreement would be overrated.

Based on our good interobserver agreement results, we suggest that prospective studies of larger series and anatomic/pathological correlation be undertaken to establish the sensitivity and specificity of CT in identifying tumor invasion of the preepiglottic space. Such studies would establish the reliability of tomographic criteria in this situation, ratifying CT as the main method for assessing the preepiglottic space.

## CONCLUSION

Results showing good reproducibility of findings reported by the observers in assessing tomographic signs suggesting tumor invasion of the preepiglottic space show that CT may be a reliable method in these cases, leading to changes in the surgical paradigm for treating laryngeal and pharyngeal cancer. In T1 and T2 staged malignancies, invasion of the preepiglottic space justifies altering an indication for surgery, either increasing the extent of surgery or opting for conservative radiotherapy.
